# Consistency of P53 immunohistochemical expression between preoperative biopsy and final surgical specimens of endometrial cancer

**DOI:** 10.3389/fonc.2023.1240786

**Published:** 2023-08-28

**Authors:** Jun Zhang, Peng Jiang, Chunxia Gong, Wei Kong, Yuan Tu, Yuzhen Huang, Ying Liu

**Affiliations:** ^1^ Department of Gynecology, People’s Hospital of Chongqing Banan District, Chongqing, China; ^2^ Department of Gynecology, The First Affiliated Hospital of Chongqing Medical University, Chongqing, China; ^3^ Department of Gynecology, Women and Children’s Hospital of Chongqing Medical University, Chongqing, China

**Keywords:** endometrial cancer, P53 immunohistochemistry, preoperative biopsy, final pathology, consistency

## Abstract

**Objective:**

The aim of this study is to explore the consistency of P53 immunohistochemical expression between preoperative biopsy and final pathology in endometrial cancer (EC), and to predict the prognosis of patients based on the 4-tier P53 expression and classic clinicopathological parameters.

**Methods:**

The medical data of patients with stage I-III EC who received preoperative biopsy and initial surgical treatment in two medical centers was retrospectively collected. The consistency of P53 immunohistochemistry expression between preoperative biopsy and final pathology was compared using Cohen’s kappa coefficient and Sankey diagram, then 4-tier P53 expression was defined (P53wt/P53wt, P53abn/P53wt, P53wt/P53abn, and P53abn/P53abn). Univariate and multivariate Cox regression analysis was used to determine the correlation between 4-tier P53 expression and the prognosis of patients. On this basis, the nomogram models were established to predict the prognosis of patients by combining 4-layer P53 expression and classic clinicopathological parameters, then risk stratification was performed on patients.

**Results:**

A total of 1186 patients were ultimately included in this study through inclusion and exclusion criteria. Overall, the consistency of P53 expression between preoperative biopsy and final pathology was 83.8%, with a kappa coefficient of 0.624. ROC curve suggested that the AUC of 4-tier P53 expression to predict the prognosis of patients was better than AUC of P53 expression in preoperative biopsy or final pathology alone. Univariate and multivariate Cox regression analysis suggested that 4-tier P53 expression was an independent influencing factor for recurrence and death. On this basis, the nomogram models based on 4-tier P53 expression and classical clinicopathological factors were successfully established. ROC curve suggested that the AUC (AUC for recurrence and death was 0.856 and 0.838, respectively) of the models was superior to the single 4-tier P53 expression or the single classical clinicopathological parameters, which could provide a better risk stratification for patients.

**Conclusion:**

The expression of P53 immunohistochemistry had relatively good consistency between preoperative biopsy and final pathology of EC. Due to the discrepancy of P53 immunohistochemistry between preoperative biopsy and final pathology, the prognosis of patients can be better evaluated based on the 4-layer P53 expression and classic clinical pathological parameters.

## Introduction

Endometrial cancer (EC) is one of the most common malignant tumors in women (1). In recent years, the incidence rate and mortality rate have been on the rise (2). EC is usually diagnosed through preoperative biopsy, and most patients have been advised to undergo surgical treatment because they are still in the early stages when they are diagnosed (3). In general, the prognosis evaluation of patients and the formulation of postoperative adjuvant treatment plans are mostly based on the final pathological results (4). In recent years, some studies have shown that certain pathological parameters such as histological types and histological grade are inconsistent between preoperative biopsy and final pathology (5). Neglecting the results of preoperative biopsy based solely on final pathology may pose a risk of inadequate evaluation of patients’ prognosis, leading to over treatment or inadequate treatment (5).

In recent years, molecular classification has shown strong prognostic value in EC, and has gradually been widely applied in clinical practice (6). The expression of P53 subgroup, which is one of the four subgroups of the molecular classification, can be easily obtained through immunohistochemistry (7). Patients with P53 wild-type (P53wt) have a relatively better prognosis, while those with P53 abnormality (P53abn) have a very poor prognosis (8). Therefore, accurate assessment of P53 expression in patients has important prognostic value. Generally speaking, there are no strict regulations for the tissues used for P53 immunohistochemistry evaluation, which can be based on preoperative biopsy tissues or final pathological specimens (7). However, at present, research on the consistency of P53 immunohistochemical expression between preoperative biopsy and final pathology of EC is still very rare. Therefore, the purpose of this study is to explore the consistency of P53 immunohistochemical expression results between preoperative biopsy and final pathology through a dual center patient cohort, and to evaluate the prognosis of patients with different P53 expression states.

## Materials and methods

### Study population

The medical data of patients with stage I-III EC who received preoperative biopsy and initial surgical treatment in the First Affiliated Hospital of Chongqing Medical University from January 2015 to May 2020, and in the Women and Children’s Hospital of Chongqing Medical University from January 2016 to May 2020 were collected, including age, body mass index (BMI), preoperative biopsy method, surgical procedures, pathological examination results (including tumor size, tumor location, tumor invasion range, histological type and grade of tumor, etc.), postoperative adjuvant treatment, and P53 immunohistochemical expression.

The inclusion criteria for patients were as follows: (1) Patients underwent preoperative biopsy and was initially diagnosed with EC; (2) Patients underwent initial surgical treatment and was ultimately diagnosed with EC by final pathology, with FIGO staging ranging from stage 1 to stage III (Most stage IV patients did not receive surgical treatment, and the assessment of recurrence in stage IV patients is difficult to determine because they have already experienced distant metastasis, so stage IV patients were not included in this study). The exclusion criteria were as follows: (1) Without standard surgery; (2) Receiving adjuvant therapy before surgery; (3) With incomplete medical records; (4) With other malignancies; (5) Lost follow-up. The study was approved by the Institutional Review Board (IRB) of the First Affiliated Hospital of Chongqing Medical University and the Women and Children’s Hospital of Chongqing Medical University (IRB number: 2021-676 and 2023-002).

### Treatment

**Figure 1:**
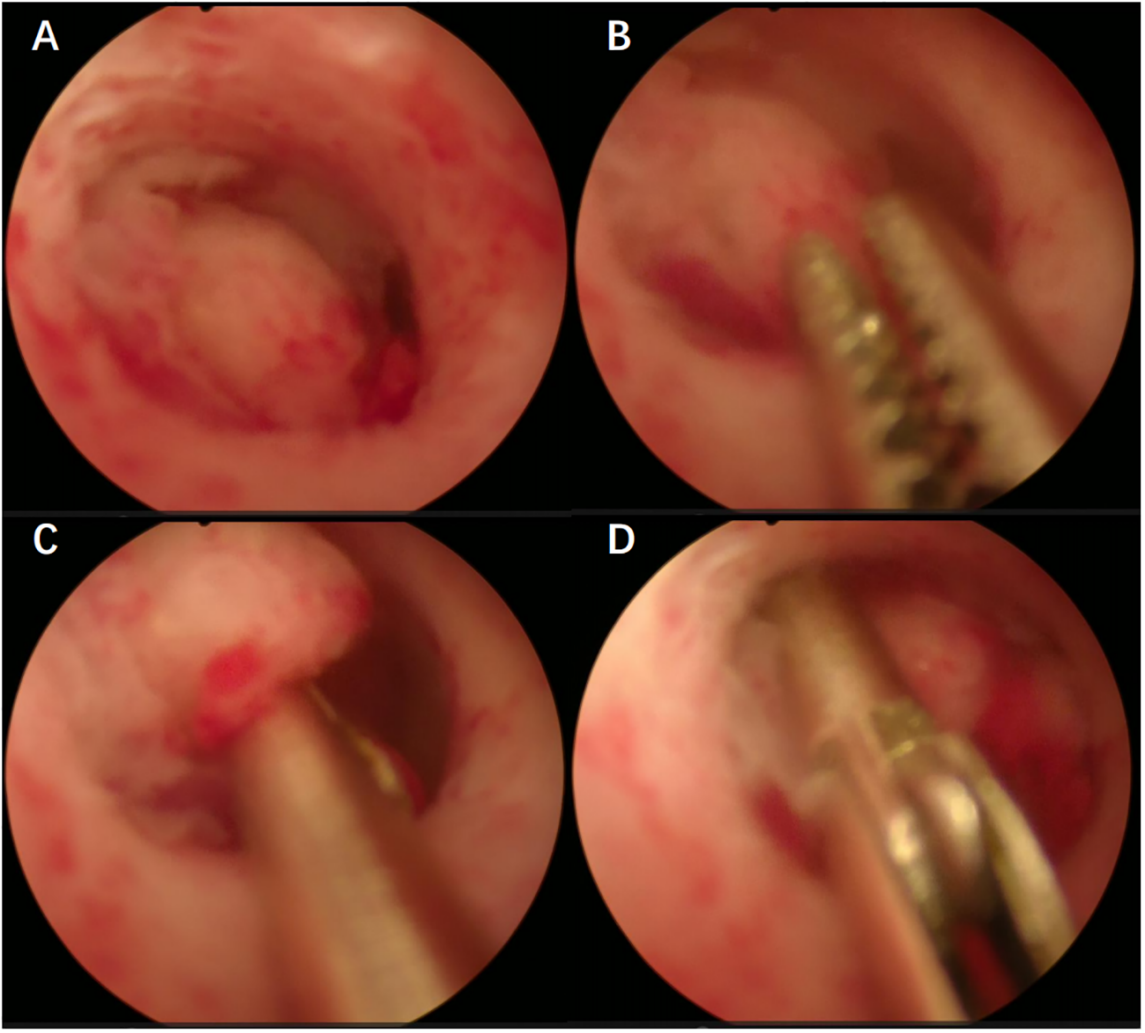
**(A–D)** Hydroscopic guided “grab” endometrial biopsy.

All patients included in this study underwent preoperative biopsy, which can be divided into two main methods: (1) Blind biopsy, including blind Division and Curettage (D&C) and Pipelle (suction biopsy); (2) Hydroscopic guided endometrial biopsy, including hydroscopic guided diagnostic curettage and hydroscopic guided “grab” endometrial biopsy (**Figure 1**). Hysteroscopic biopsy is performed by professional hysteroscopic physicians at their respective medical centers. The patient subsequently underwent a comprehensive staged surgery that included at least abdominal total hysterectomy + bilateral salpingo-oophorectomy + pelvic lymph node dissection ± para-aortic lymph node dissection (3). After surgery, it was recommended that patients receive corresponding adjuvant treatment according to international guidelines and multidisciplinary discussions (3, 9). The specific adjuvant treatment plans refer to similar previous studies (10, 11).

### Follow-up

Follow-up was performed every 3 months for the first 2 years after surgery, every 6 months for the next 3 years, and annually thereafter (3). The follow-up plan included regular physical examinations and necessary auxiliary examinations. The follow-up deadline for this study was May 2023, and each patient has been guaranteed a follow-up period of more than 3 years. Two or more gynecological oncologists confirmed recurrence through physical examination, biochemical indicators, imaging examination, and pathological biopsy (10). Recurrence included vaginal stump recurrence, central pelvic region recurrence, upper para-aortic lymph node metastases, peritoneal metastases, and metastases to other organs (11). Recurrence-free survival (RFS) was defined as the time from the surgical date to the confirmed recurrence date, and overall survival (OS) was defined as the time from the surgical date to the death (10).

### Pathological analysis and P53 immunohistochemistry analysis

Preoperative biopsy tissues and postoperative surgical specimens of patients were immediately fixed with standard 10% neutral formalin tissue fixative after being removed from the body (the entire process usually does not exceed 10 minutes). The specimens were completely immersed in the fixative (the volume of the fixative is usually more than 5 times that of the specimens), the fixing time was between 24 hours and 48 hours. Finally, the specimens were sent to the Pathology Experimental Center of Chongqing Medical University for subsequent processing (dehydration, paraffin embedding, sectioning, H&E staining, and immunohistochemical analysis) within 24 hours. The evaluation of pathological results (including tumor size, histological type and grade, depth of myometrial invasion, cervical stromal invasion, LVSI, lymph node involvement, etc.) were performed by professional pathologists. The pathological type I of EC was defined as G1 and G2 endometrioid adenocarcinoma, while type II was defined as G3 endometrioid adenocarcinoma and non-endometrioid adenocarcinoma, including serous carcinoma, clear cell carcinoma, and other special histological types (12).

According to a unified and optimized immunohistochemical protocol, immunohistochemical analysis of P53 protein was performed on an immunohistochemical automatic staining machine (Leica Bond Max, Milton Keynes, UK). P53 antibody (MAB-0674, Maixin Biotech, China) was used as a primary antibody for immunohistochemical analysis of P53 (specific immunohistochemical steps can be found in references) (10, 13).

**Figure 2:**
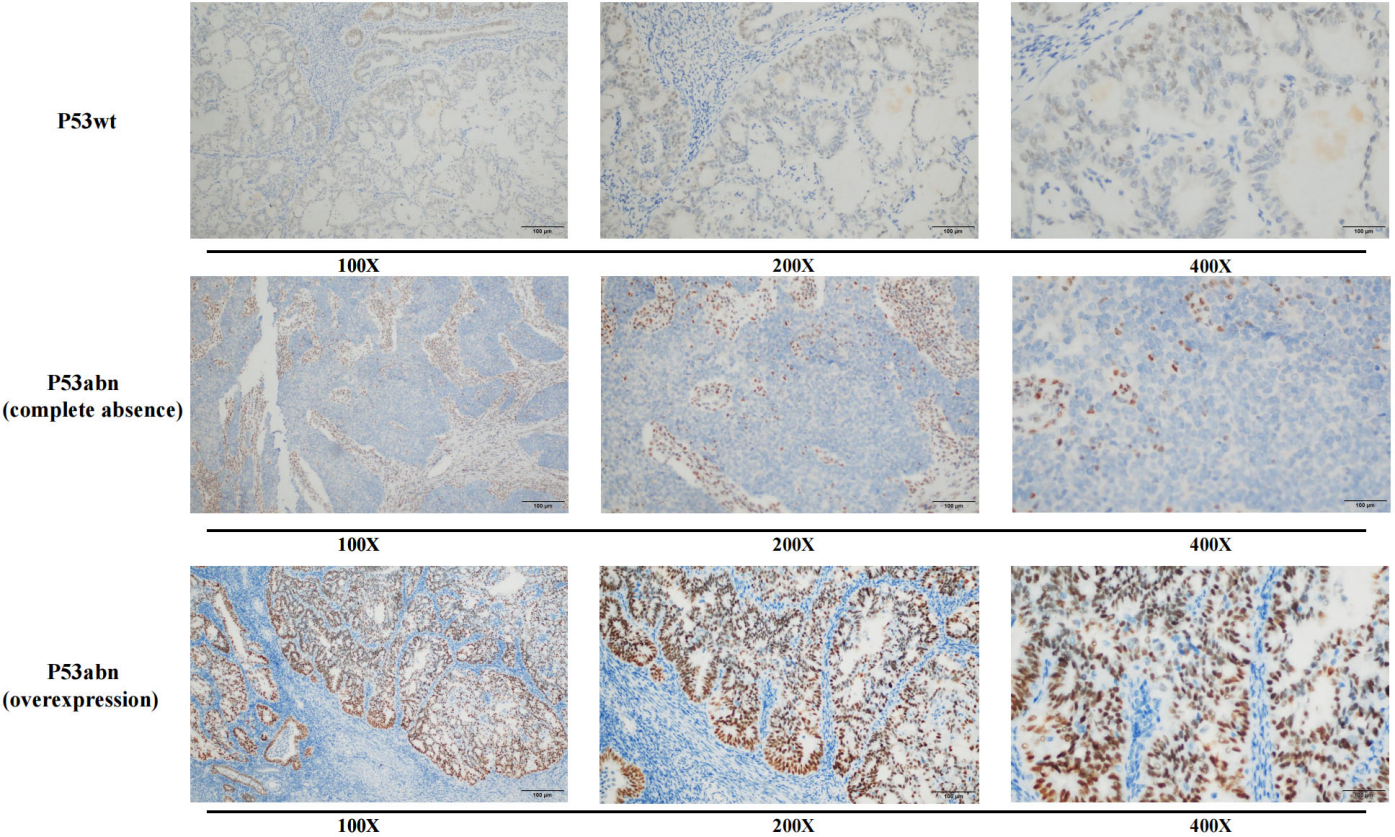
Immunohistochemical expression of P53wt and P53abn.

The immunohistochemical results of P53 were evaluated based on the staining intensity (weak staining, medium staining, and strong staining) and the proportion of positive cells. According to the criteria of P53 immunohistochemical interpretation, overexpression (generally >75% strongly positive staining) or complete loss of expression (no significantly positive tumor cells) of P53 was defined as P53 abnormality (P53abn), while positive expression between these two extremes was defined as P53 wild-type (P53wt) expression (**Figure 2**) (14, 15).

### Definition of 4-tier P53 expression

For the convenience of subsequent research, based on the different expression states of P53 in preoperative biopsy and final pathology, 4-tier P53 expression was defined: P53 immunohistochemistry showed P53wt expression in both preoperative biopsy and final pathology was defined as P53wt/P53wt; Similarly, P53 immunohistochemistry showed P53abn expression in both preoperative biopsy and final pathology was defined as P53abn/P53abn; P53 immunohistochemistry showed P53wt expression in preoperative biopsy while showed P53abn expression in final pathology was defined as P53wt/P53abn; P53 immunohistochemistry showed P53abn expression in preoperative biopsy while showed P53wt expression in final pathology was defined as P53abn/P53wt. In order to reduce subjective interpretation errors, a secondary review was conducted by a professional pathologist for cases with differences in P53 between preoperative biopsy and final pathology in this study.

### Experimental design and statistical analysis

Firstly, the Cohen’s kappa coefficient and Sankey diagram were used to compare the consistency of P53 immunohistochemistry results between preoperative biopsy and final pathology (16). The kappa coefficient (κ) could be explained as follows: κ <0.01 indicated no consistency, 0.01-0.20 indicated slight consistency, 0.21-0.40 indicated general consistency, 0.41-0.60 indicated moderate consistency, 0.61-0.80 indicated substantial consistency, and 0.81-1.00 indicated almost complete consistency (17). The prognostic value of 4-tier P53 expression, P53 expression in preoperative biopsy and P53 expression in final pathology was compared by using receiver operating characteristic (ROC) curve and area under the curve (AUC). Univariate and multivariate Cox regression analysis was used to determine the correlation between 4-tier P53 expression and the prognosis of patients. On this basis, the nomogram models for predicting patient prognosis were established by combining 4-tier P53 expression and classic clinicopathological parameters, and the models were validated using calibration curves. ROC curve and the maximum value of Youden index (Youden index = sensitivity +specificity -1) were used to determine the optimal risk threshold of the models and then risk stratification was performed on patients (18). Kaplan Meier analysis and log-rank test were used to compare the prognosis between patients in high-risk group and in non-high-risk group patients, and stratified analysis based on different adjuvant treatment methods was further conducted. Categorical variable was expressed in frequency (%), continuous variable of normal distribution was expressed in mean (± SD), and continuous variable of non-normal distribution was expressed in median (P25, P75). SPSS software (version 25.0, IBM Statistics, Chicago, IL, USA) and R software (version 4.0.3, http://www.r-project.org) were used for data analysis.

## Results

### Baseline data of patients

**Figure 3:**
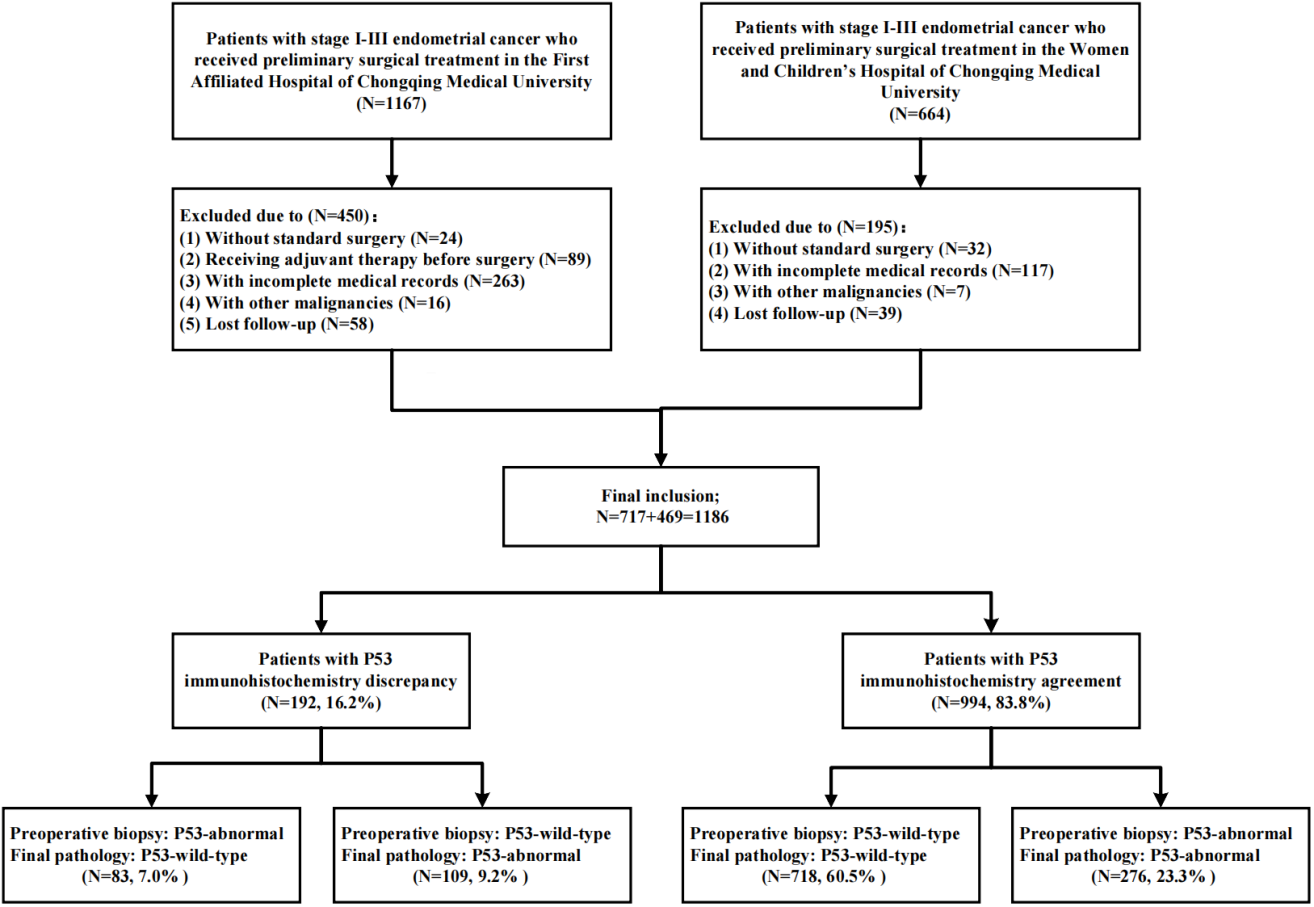
Flow chart of patient inclusion.

As shown in **Figure 3**, a total of 1186 patients were included in this study through inclusion and exclusion criteria. The baseline data of the patients was shown in **Table 1**. The average age of the patients was 53.67 (± 9.28) years old, of which 845 (71.2%) patients were with FIGO stage I and 185 (15.6%) patients had lymph node metastasis (LNM). A total of 737 (62.1%) patients received adjuvant therapy after surgery, of which 389 (32.8%) patients received radiotherapy, 309 (26.1%) patients received chemoradiotherapy, and 39 (3.3%) patients only received chemotherapy due to personal reasons. The median follow-up time of the patients was 44.00 (34.00, 61.00) months, and a total of 183 (15.4%) patients experienced recurrence during the follow-up period. The main form of recurrence was distant metastasis (32.2%), followed by recurrence in the central pelvic region (28.4%); A total of 139 (11.7%) patients died, of which 132 (11.1%) died due to recurrence.

**Table 1:** Baseline characteristics of patients.

Variable	All patients (N = 1186)
**Age [yrs, mean (± SD)]**	53.67 ( ± 9.28)
**BMI [kg/m^2^, mean (± SD)]**	24.57 ( ± 3.68)
FIGO staging	
I	845 (71.2%)
II	101 (8.6%)
III	240 (20.2%)
Pathological type	
**-Preoperative biopsy**	
I	830 (70.0%)
II	356 (30.0%)
**-Final pathology**	
I	849 (71.6%)
II	337 (28.4%)
Myometrial invasion	
<1/2	818 (69.0%)
≥1/2	368 (31.0%)
Cervical stromal invasion	
No	997 (84.1%)
Yes	189 (15.9%)
LVSI	
Negative	870 (73.4%)
Positive	316 (26.6%)
Lymph node metastasis	
No	1001 (84.4%)
Yes	185 (15.6%)
P53 expression	
**-Preoperative biopsy**	
P53wt	827 (69.7%)
P53abn	359 (30.3%)
**-Final pathology**	
P53wt	801 (67.5%)
P53abn	385 (32.5%)
Preoperative sampling method	
D&C or Pipelle biopsy	652 (55.0%)
Hysteroscopic biopsy	534 (45.0%)
Adjuvant treatment	
Follow-up	449 (37.9%)
Only radiotherapy	389 (32.8%)
Only chemotherapy	39 (3.3%)
Chemoradiotherapy	309 (26.1%)
Recurrence	
No	1003 (84.6%)
Yes	183 (15.4%)
Sites of relapsed (n=183)	
Vaginal stump	11 (6.0%)
Central pelvic region	52 (28.4%)
Lymph nodes (upper para-aortic)	21 (11.5%)
Peritoneal metastases	40 (21.9%)
Metastasis to other organs	59 (32.2%)
Death	
Death of recurrence	132 (11.1%)
Death of other reasons	7 (0.6%)
Alive	1047 (88.3%)
**RFS time [months, median (P25, P75)]**	42.00 (31.00, 60.00)
**Follow-up [months, median (P25, P75)]**	44.00 (34.00, 61.00)

BMI, body mass index; FIGO, International Federation of Gynecology and Obstetrics; LVSI, lymphatic vessel space invasion; P53abn, P53 abnormality; P53wt, P53 wild-type; D&C, dilation and curettage; RFS, recurrence-free survival.

534 (45.0%) patients obtained tissue samples through hysteroscopic guided biopsy before surgery, while the remaining patients obtained tissue samples through D&C or Pipelle biopsy before surgery. Overall, 830 (70.0%) EC patients were assessed as pathological type I through preoperative biopsy, while 849 (71.6%) EC patients were confirmed as pathological type I in the final pathology. Similarly, 359 (30.3%) and 385 (32.5%) patients were diagnosed as P53abn expression in preoperative biopsy and final pathology, respectively.

### Consistency analysis of P53 immunohistochemical expression in preoperative biopsy and final examination

**Figure 4:**
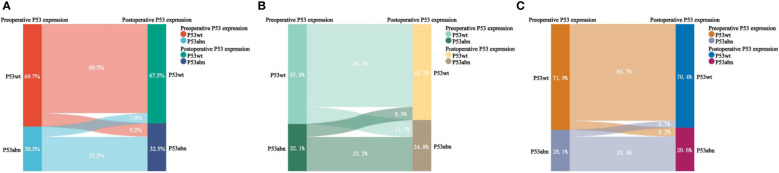
Sankey diagram of P53 expression in preoperative biopsy and final pathology. **(A)** For all patients; **(B)** For patients with blind biopsy (D&C and Pipelle); **(C)** For patients with hydroscopic guided endometrial biopsy.

As shown in **Table 2** and **Figure 4**, there was inconsistency in the expression of P53 immunohistochemistry between preoperative biopsy and final pathology. Overall, 9.2% of patients diagnosed as P53wt in preoperative biopsy were diagnosed as P53abn in final pathology, while 7.0% of patients diagnosed as P53abn in preoperative biopsy were diagnosed as P53wt in final pathology. The consistency of P53 expression between preoperative biopsy and final pathology was 83.8%, with a kappa coefficient of 0.624. Layered analysis of preoperative biopsy methods revealed that the consistency of P53 expression between preoperative specimens obtained through hysteroscopic biopsy and final pathology was 89.1%, with a kappa coefficient of 0.735. While the consistency of P53 expression between the preoperative specimens obtained through blind biopsy (D&C or Pipelle biopsy) and the final pathological results was relatively low (79.4%), with a kappa coefficient of 0.539.

**Table 2:** Consistency of P53 expression between preoperative biopsy and final pathology.

Preoperative biopsy	Final pathology		
Total (n=1186, overall agreement: 83.8%, kappa: 0.624)			
	P53wt	P53abn	Total
P53wt	718 (60.5%)	109 (9.2%)	827 (69.7%)
P53abn	83 (7.0%)	276 (23.3%)	359 (30.3)
Total	801 (67.5%)	385 (32.5%)	1186 (100%)
D&C or Pipelle biopsy (n=652, overall agreement: 79.4%, kappa: 0.539)			
	P53wt	P53abn	Total
P53wt	367 (56.3%)	76 (11.7%)	443 (67.9%)
P53abn	58 (8.9%)	151 (23.2%)	209 (32.1%)
Total	425 (65.2%)	227 (34.8%)	652 (100%)
Hysteroscopic biopsy (n=534, overall agreement: 89.1%, kappa: 0.735)			
	P53wt	P53abn	Total
P53wt	351 (65.7%)	33 (6.2%)	384 (71.9)
P53abn	25 (4.7%)	125 (23.4%)	150 (28.1%)
Total	376 (70.4%)	158 (29.6%)	534 (100%)

P53abn, P53 abnormality; P53wt, P53 wild-type.

It was worth noting that we also found a strong correlation between the consistency of P53 and the consistency of pathological type. As shown in **Supplementary Table 1**, in the group of patients with consistent P53 expression (n=994), 900 patients (90.5%) had consistent pathological type between preoperative biopsy and final pathology. In the group of patients with inconsistent P53 expression (n=192), 133 patients (69.3%) also showed differences in pathological type (correlation P value<0.001).

### Comparison of the accuracy of P53 expression under different scenarios for predicting recurrence and death of EC

**Figure 5:**
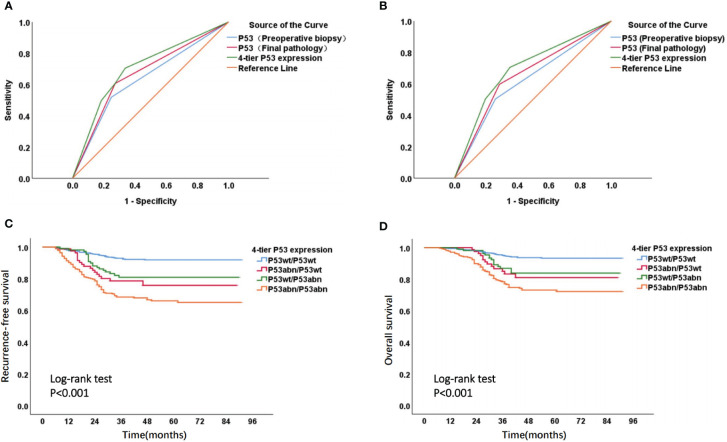
ROC curve and Kaplan-Meier survival curve of 4-tier P53 expression. ROC curve of P53 expression in different states to predict recurrence **(A)** and death **(B)** of patients; Kaplan Meier survival curve of RFS **(C)** and OS **(D)** in four subgroups of 4-tier P53 expression.

The ROC curve suggested that, for the prediction of EC recurrence and death, the AUC (AUC for recurrence and death was 0.702 and 0.695, respectively) of 4-tier P53 expression, which was defined by the combination of P53 expression in preoperative biopsy and P53 expression in final pathology, was superior to the AUC of P53 expression in simple preoperative biopsy (AUC for recurrence and death was 0.635 and 0.621) or the AUC of P53 expression in simple final pathology (AUC for recurrence and death was 0.667 and 0.655) (**Figures 5A, B**). The Kaplan Meier analysis (**Figures 5C, D**) suggested significant survival differences among the four groups of 4-tier P53 expression. The P53wt/P53wt group had the best prognosis (RFS and OS), the P53abn/P53abn group had the worst prognosis, while the P53wt/P53abn group and P53abn/P53wt group had similar prognosis, the prognosis of these two groups was between the P53wt/P53wt group and the P53bn/P53bnn group. The specific prognosis of each group was shown in **Supplementary Table 2**.

### Univariate and multivariate Cox regression analysis related to prognosis (RFS and OS) of EC

Univariate and multivariate Cox regression analysis related to RFS and OS was conducted, respectively (**Tables 3**, **4**). Univariate Cox regression analysis found that multiple clinicopathological factors, including age, FIGO stage, pathological type in final pathology, myometrial invasion, cervical stromal invasion, LVSI, lymph node metastasis, and 4-tier P53 expression, were significantly correlated with RFS and OS. Factors with a P value < 0.05 in univariate analysis were further included in multivariate analysis. The results of multivariate analysis showed that only 6 factors were both significantly correlated with the RFS and OS, namely FIGO stage, pathological type in final pathology, myometrial invasion, LVSI, lymph node metastasis, and 4-tier P53 expression.

**Table 3:** Univariate and multivariate Cox regression analysis of RFS of EC.

Variables	Univariate analysis			Multivariate analysis		
	Hazard ratio	95% CI	P-value	Hazard ratio	95% CI	P-value
**Age** (≥60 vs <60)	1.513	1.108-2.066	0.009	1.115	0.806-1.544	0.511
**BMI**	1.009	0.970-1.049	0.669			
FIGO stage						
I	ref		<0.001	ref		0.011
II	2.265	1.285-3.993	0.005	1.910	0.954-3.824	0.068
III	8.753	6.368-12.032	<0.001	2.448	1.345-4.454	0.003
**Pathological type in final pathology** (Type II vs Type I)	3.737	2.790-5.006	<0.001	2.015	1.466-2.768	<0.001
**Myometrial invasion** (≥1/2 vs <1/2)	2.826	2.113-3.778	<0.001	1.532	1.104-2.124	0.011
**Cervical stromal invasion** (Yes vs No)	2.723	1.987-3.730	<0.001	1.048	0.699-1.570	0.820
**LVSI** (Positive vs Negative)	3.487	2.607-4.662	<0.001	1.893	1.369-2.618	<0.001
**Lymph node metastasis** (Yes vs No)	8.123	6.067-10.874	<0.001	2.151	1.284-3.604	0.004
**Adjuvant treatment** (Yes vs No)	1.472	1.073-2.020	0.016	0.754	0.534-1.065	0.109
4-tier P53 expression						
P53wt/P53wt	ref		<0.001	ref		<0.001
P53abn/P53wt	3.233	1.896-5.513	<0.001	2.666	1.550-4.583	<0.001
P53wt/P53abn	2.544	1.523-4.250	<0.001	1.713	1.008-2.912	0.047
P53abn/P53abn	5.001	3.571-7.005	<0.001	3.506	2.464-4.990	<0.001

BMI, body mass index; FIGO, International Federation of Gynecology and Obstetrics; LVSI, lymphatic vessel space invasion; P53wt/P53wt, P53 wild-type in both preoperative biopsy and final pathology; P53abn/P53wt, P53 abnormality in preoperative biopsy while P53 wild-type in final pathology; P53wt/P53abn, P53 wild-type in preoperative biopsy while P53 abnormality in final pathology; P53abn/P53abn, P53 abnormality in both preoperative biopsy and final pathology.

**Table 4:** Univariate and multivariate Cox regression analysis of OS of EC.

Variables	Univariate analysis			Multivariate analysis		
	Hazard ratio	95% CI	P-value	Hazard ratio	95% CI	P-value
**Age** (≥60 vs <60)	1.552	1.088-2.215	0.015	1.082	0.748-1.566	0.675
**BMI**	1.024	0.979-1.070	0.300			
FIGO stage						
I	ref		<0.001	ref		0.012
II	2.563	1.349-4.869	0.004	1.877	0.860-4.095	0.114
III	8.921	6.159-12.922	<0.001	2.779	1.412-5.472	0.003
**Pathological type in final pathology** (Type II vs Type I)	3.117	2.233-4.350	<0.001	1.467	1.015-2.119	0.041
**Myometrial invasion** (≥1/2 vs <1/2)	3.026	2.166-4.229	<0.001	1.562	1.078-2.263	0.019
**Cervical stromal invasion** (Yes vs No)	3.003	2.109-4.277	<0.001	1.118	0.713-1.752	0.627
**LVSI** (Positive vs Negative)	3.136	2.248-4.374	<0.001	1.572	1.085-2.275	0.017
**Lymph node metastasis** (Yes vs No)	7.551	5.408-10.543	<0.001	1.873	1.045-3.355	0.035
**Adjuvant treatment** (Yes vs No)	1.230	0.865-1.748	0.249			
4-tier P53 expression						
P53wt/P53wt	ref		<0.001	ref		<0.001
P53abn/P53wt	3.045	1.631-5.683	<0.001	2.335	1.245-4.381	0.008
P53wt/P53abn	2.504	1.386-4.524	0.002	1.941	1.053-3.579	0.034
P53abn/P53abn	4.773	3.246-7.018	<0.001	3.386	2.254-5.086	<0.001

BMI, body mass index; FIGO, International Federation of Gynecology and Obstetrics; LVSI, lymphatic vessel space invasion; P53wt/P53wt, P53 wild-type in both preoperative biopsy and final pathology; P53abn/P53wt, P53 abnormality in preoperative biopsy while P53 wild-type in final pathology; P53wt/P53abn, P53 wild-type in preoperative biopsy while P53 abnormality in final pathology; P53abn/P53abn, P53 abnormality in both preoperative biopsy and final pathology.

### Predicting prognosis of EC by combining the 4-tier P53 expression with classic clinicopathological factors

**Figure 6:**
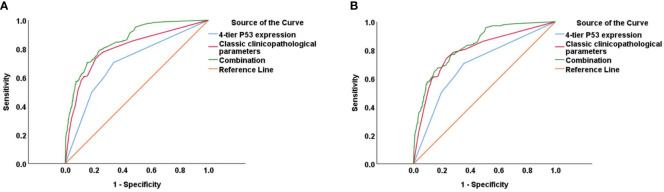
The ROC curve of 4-tier P53 expression, classic clinicopathological parameters, and their combination (4-tier P53 expression+ classic clinicopathological parameters) for predicting the recurrence **(A)** and death **(B)** of EC.

**Figure 7:**
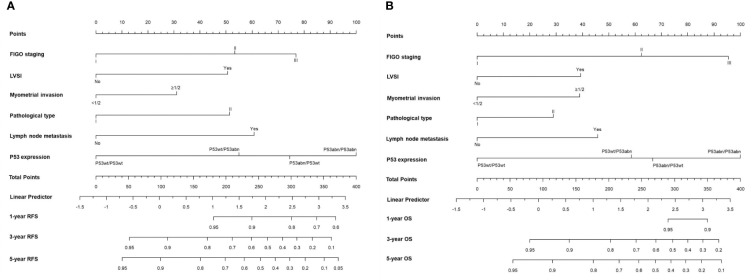
The two nomogram models for predicting RFS **(A)** and OS **(B)** of EC patients based on 4-tier P53 expression and classical clinicopathological parameter.

**Figure 8:**
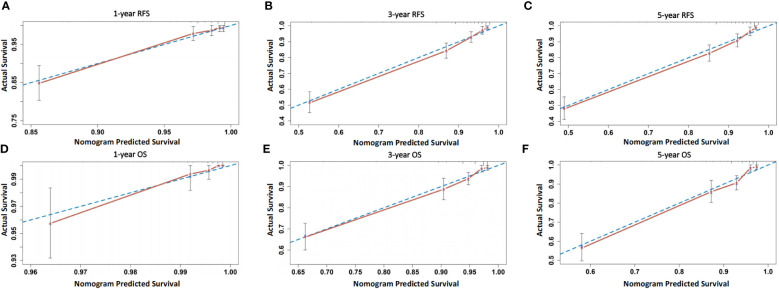
Calibration curves of the two models. **(A–C)** Calibration curves of the model for predicting 1-, 3-, and 5-year RFS of patients; **(D–F)** Calibration curves of the model for predicting 1-, 3-, and 5-year OS of patients.

The prognostic value of using 4-tier P53 expression alone to predict patient prognosis was still very limited. Based on this, we compared the AUC of 4-tier P53 expression, classic clinicopathological parameters, and their combination (4-tier P53 expression+ classic clinicopathological parameters) for predicting the recurrence and death of EC. The ROC curve showed that the AUC of the combination for predicting the EC recurrence and death were 0.856 (95% CI, 0.828-0.885) and 0.838 (95% CI, 0.804-0.871), respectively, which was higher than the AUC of simple clinicopathological parameters or the AUC of simple 4-tier P53 expression (**Supplementary Table 3** and **Figure 6**).Therefore, based on the results of multivariate analysis, we combined the 4-tier P53 expression with classic clinicopathological factors to construct the nomogram models for predicting EC recurrence and death, respectively. Based on these two models, personalized predictions can be made for the 1-year, 3-year, 5-year RFS and OS rates of patients (**Figure 7**). The calibration curves related to RFS and OS showed good fitness of these two models (**Figure 8**).

**Figure 9:**
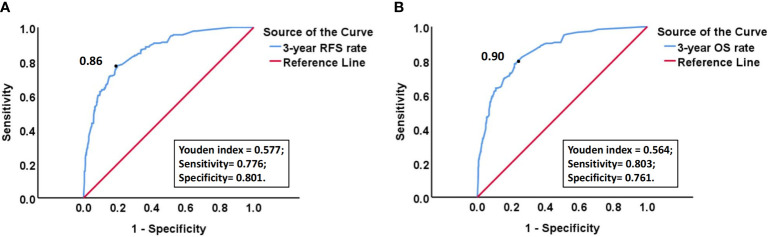
**(A)** ROC curve of 3-year RFS rate calculated by the model for predicting the recurrence of EC; **(B)** ROC curve of 3-year OS rate calculated by the model for predicting the death of EC. “Black dot” indicates that at this point, Youden index is the largest, so the probability corresponding to this point is the optimal risk threshold of each model.

**Figure 10:**
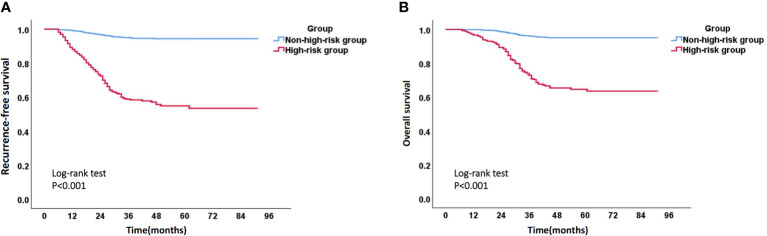
Kaplan-Meier survival curve of high-risk group and non-high-risk group. **(A)** Recurrence-free survival curve of high-risk group and non-high-risk group; **(B)** Overall survival curve of high-risk group and non-high-risk group.

Due to the fact that most recurrent patients of EC experience recurrence within 3 years after surgery, we calculated the 3-year RFS rate, as well as the corresponding sensitivity and specificity, for each patient using the above model. ROC curve and the maximum value (0.577) of Youden index (Youden index= sensitivity +specificity -1) showed that the optimal risk threshold of the 3-year RFS rate of the model was 0.86 (corresponding sensitivity was 0.776, specificity was 0.801) (**Figure 9A**). Similarly, the optimal risk threshold for the 3-year OS rate of another model was 0.90 (corresponding sensitivity was 0.803, specificity was 0.761) (**Figure 9B**). Then patients with a 3-year RFS rate < 0.86 or a 3-year OS rate < 0.90 were defined as high-risk group, while the remaining patients were defined as non-high-risk group. The Kaplan Meier analysis suggested that the RFS and OS rates of high-risk group were significantly lower than those of non-high-risk group (**Figure 10** and **Supplementary Table 4**).

**Figure 11:**
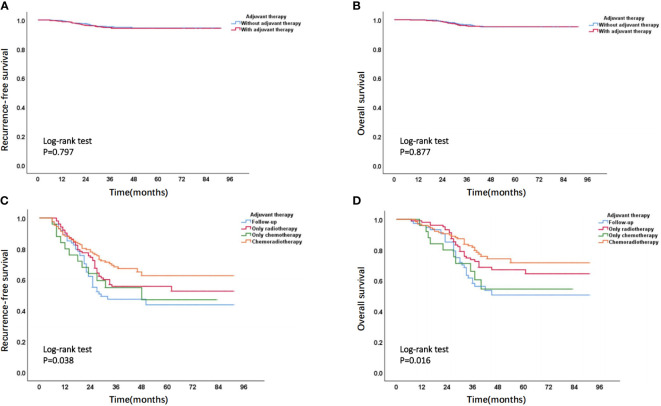
**(A)** Recurrence-free survival curve and **(B)** overall survival curve of patients with or without adjuvant therapy in non-high-risk group; **(C)** Recurrence-free survival curve and **(D)** overall survival curve of patients receiving different adjuvant treatment methods in high-risk group.

Based on stratified analysis of adjuvant therapy, it was found that, in the non-high-risk group, there was no significant difference in prognostic outcomes between patients who received adjuvant therapy and those who did not receive adjuvant therapy (**Figures 11A, B**). In high-risk group, the overall prognosis of patients who received adjuvant treatment was to varying degrees better than those who did not receive adjuvant treatment, and the prognosis of patients receiving chemoradiotherapy was better than that of patients receiving chemotherapy or radiotherapy alone (**Figures 11C, D**). The specific prognosis of patients who received different adjuvant treatment methods in high-risk group was shown in **Supplementary Table 5**.

## Discussion

Traditionally, discussions on the consistency between preoperative biopsy and final pathology of EC have mainly focused on pathological parameters such as histological type or grade (4). In recent years, molecular markers, especially molecular classification, have been gradually popularized in clinical applications in EC due to their prognostic value independent of classical pathological parameters (6). P53abn is the molecular subgroup with the worst prognosis among the four subgroups of molecular classification, which has important prognostic value and therapeutic significance (8). Compared to gene sequencing, the expression results of P53 can be easily obtained through immunohistochemistry, but research on the consistency of P53 immunohistochemistry expression between preoperative biopsy and final pathology is still very rare (7).

In this study, we preliminarily explored the consistency of P53 immunohistochemical expression between preoperative biopsy and final pathology by including a dual center patient cohort. The results showed that the expression of P53 immunohistochemistry had a relatively high consistency between preoperative biopsy and final pathology, and the consistency of biopsy guided by hysteroscopy was higher than that of blind biopsy. The advantages of hysteroscopic biopsy have also been demonstrated in other similar studies. In Attilio et al.’s study, it has been reported that hysteroscopic guided endometrial biopsy can more accurately diagnose the histological type and grade of EC (19). This is not difficult to understand, compared to blind biopsy, hysteroscopic guided endometrial biopsy can sample target areas of the endometrium (such as suspected cancerous areas) under direct vision (17). Therefore, in conditional medical centers, preoperative use of hysteroscopy for endometrial biopsy can be an effective alternative to blind biopsy.

In fact, we believe that the main reason for the differential expression between preoperative biopsy and final pathology, in addition to subjective interpretation errors, is tumor heterogeneity, that is, within a tumor range, all tumor cells and tissues do not have a single tumor feature, but rather have their own relatively specific different characteristics and clinical manifestations (19, 20). The main results for supporting the above conjecture are as follows: firstly, the consistency between preoperative biopsy and final pathology mentioned earlier is related to the preoperative biopsy method, the consistency of biopsy guided by hysteroscopy is higher than that of blind biopsy; Secondly, our results also found a strong correlation between the consistency of P53 expression and the consistency of histological type. In the group of patients with consistent P53 expression between preoperative biopsy and final pathology, the proportion of patients with consistent histological types between preoperative biopsy and final pathology was higher. Similarly, in the group of patients with inconsistent P53 expression, the proportion of patients with inconsistent histological types was also higher (**Supplementary Table 1**).

Of course, regardless of the cause, the discrepancy between preoperative biopsy and final pathology is a fact. In terms of P53 expression, our research results found that patients in different groups of 4-tier P53 expression had different prognosis outcomes. The P53wt/P53wt group had the best prognosis, while the P53abn/P53abn group had the worst prognosis, which is not difficult to understand. However, we need to pay special attention to the poor prognosis of patients in the P53abn/P53wt group and the P53wt/P53abn group. This suggested that relying solely on preoperative biopsy results or final pathological results for prognosis evaluation of patients may pose a risk of insufficient diagnosis, especially for patients with preoperative blind biopsy, as the consistency between preoperative biopsy and final pathology was relatively low. Therefore, it was more necessary to combine preoperative biopsy results and final pathological results for comprehensive evaluation of patients (5). Further ROC curve also suggested that the AUC of 4-tier P53 expression was better than that of P53 expression in preoperative biopsy alone or that of P53 expression in final pathology alone (**Figures 5A, B**), which undoubtedly confirmed our above statement.

In order to further improve the accuracy of prognostic evaluation for EC patients, we constructed two nomogram models by combining with 4-tier P53 expression and classic clinicopathological parameters to predict EC recurrence and death. The ROC curve suggested that the prediction performance of these two models was better than that of the simple clinicopathological parameters or the simple 4-tier P53 expression. At the same time, the overall prognosis of patients in high-risk group divided by the two models was much lower than that of patients in the non-high-risk group, indicating that the two models can perform better risk stratification for patients. In the latest ESMO guidelines, a new prognostic risk group has been proposed based on molecular classification and classic clinicopathological parameters, which undoubtedly coincides with our philosophy (21). It was worth mentioning that patients in high-risk group who received adjuvant treatment had a trend towards survival benefits, and the patients who received chemoradiotherapy had the greatest survival benefits (**Figures 11C, D** and **Supplementary Table 5**). This may be because most patients in high-risk group were accompanied with advanced FIGO stage, poor histological type, or poor molecular subgroup such as P53abn. In the PORTEC-3 trial and subsequent studies, the latest data showed that the chemoradiotherapy group had a significant survival benefit compared to the radiotherapy group (22). In subgroup analysis, it was found that patients with stage III, serous histological type, and P53abn subgroup had the greatest survival benefit, which was similar to our research findings (22, 23). This suggested that for the high-risk group classified by the models, if conditions permit, it was recommended that patients should receive adjuvant treatment as much as possible, and the adjuvant treatment should mainly be chemoradiotherapy (21, 24).

It is worth mentioning that, in our study, 106 patients underwent gene sequencing to determine the status of TP53 (21 patients with TP53 mutations and 85 patients with TP53 wild-type). Among these 106 patients, the consistency between the immunohistochemistry results of P53 in preoperative biopsy and the results of genetic testing was 87.7% (93 patients were consistent, 13 patients were inconsistent), the consistency between the immunohistochemistry results of P53 in final pathology and the results of genetic testing was 90.6% (96 patients were consistent, 10 patients were inconsistent) (data not shown). Therefore, there is indeed inconsistency between the immunohistochemistry results and genetic testing results of P53, but overall consistency between them is still high. Similarly, Naveena Singh et al. reported a high consistency of 92.1% between immunohistochemistry results and sequencing results of P53 (7). Of course, although the immunohistochemistry results and genetic testing results of P53 are highly consistent, for cases where the immunohistochemistry results of P53 between preoperative biopsy and final pathology are inconsistent, we encourage further genetic testing to determine the status of TP53 mutations if conditions permit. In this situation, we believe that reinterpreting immunohistochemistry results based on molecular results is necessary and more convincing.

The biggest advantage of this study lied in the inclusion of a dual center patient cohort, with a sufficiently large sample size. The consistency of P53 immunohistochemical expression was explored based on different preoperative biopsy methods, and the combination of 4-tier P53 expression and classic clinicopathological parameters was proposed to predict patient prognosis. Of course, this study also had certain limitations. First, this study was a retrospective study and need to be verified by prospective cohort study. Secondly, the models established in this study also required external validation for clinical promotion and application.

## Conclusion

In a word, the expression of P53 immunohistochemistry showed relatively good consistency between preoperative biopsy and final pathology of EC. Hysteroscopic guided biopsy can improve the consistency of P53 expression between preoperative biopsy and final pathology compared to blind biopsy. Due to the discrepancy of P53 between preoperative biopsy and final pathology, the prognosis of patients can be better evaluated based on 4-tier P53 expression and classic clinicopathological parameters.

## Data availability statement

The original contributions presented in the study are included in the article/**Supplementary Material**, further inquiries can be directed to the corresponding author.

## Ethics statement

The studies involving humans were approved by the Institutional Review Board (IRB) of the First Affiliated Hospital of Chongqing Medical University and the Women and Children’s Hospital of Chongqing Medical University (IRB number: 2021-676 and 2023-002). The studies were conducted in accordance with the local legislation and institutional requirements. Written informed consent for participation in this study was provided by the participants’ legal guardians/next of kin.

## Author contributions

YL: Conceptualization, Methodology, Writing - Review & Editing. JZ: Methodology, Data curation, Investigation, Software, Formal analysis, Writing- Original draft preparation, Writing - Review & Editing. PJ: Data curation, Investigation, Writing- Original draft preparation, Writing - Review & Editing. CG, WK, YT, YH: Data curation, Supervision. All authors critically reviewed the paper and approved the final version.
