# Supplementary material for: Consistency of P53 immunohistochemical expression between preoperative biopsy and final surgical specimens of endometrial cancer
Source: Front Oncol. 2023 Aug 28;13:1240786. doi: 10.3389/fonc.2023.1240786 (PMC10493386; doi:10.3389/fonc.2023.1240786)
Supplement: Supplementary file 1 [file DataSheet_1.docx]

**Supplementary table 1**

**The correlation between the consistency of pathological type and the consistency of P53 expression**

| **Variable** | **P53 expression between preoperative biopsy and final pathology** | | **P-value*** |
| --- | --- | --- | --- |
|  | Discrepancy  (n=192) | Agreement  (n=994) |  |
| **Pathological type between preoperative biopsy and final pathology** |  |  |  |
| Discrepancy | 133 (69.3%) | 94 (9.5%) | <0.001 |
| Agreement | 59 (30.7%) | 900 (90.5%) |  |
| **Notes:** P-value*, the P-value of the correlation between the consistency of pathological type and the consistency of P53 expression. | | | |

**Supplementary table** **2**

**Analysis of survival differences among 4 subgroups of 4-tier P53 expression**

| 4-tier P53 expression | Number of recurrences  (n=183) | 3-year RFS rate (95%CI) | 5-year RFS rate  (95% CI) | P-value ^a^ | Number of deaths  (n=139) | 3-year OS rate  (95%CI) | 5-year OS rate  (95%CI) | P-value ^b^ |
| --- | --- | --- | --- | --- | --- | --- | --- | --- |
| P53wt/P53wt  (n=718, 60.5%) | 54 | 92.90%  (90.94%-94.86%) | 91.90%  (89.74%-94.06%) | <0.001 | 41 | 95.10%  (93.53%-96.67%) | 93.30%  (91.34%-95.26%) | <0.001 |
| P53abn/P53wt  (n=83, 7.0%) | 18 | 78.60%  (69.58%-87.62%) | 75.80%  (65.61%-85.99%) |  | 13 | 85.00%  (76.77%-93.23%) | 81.00%  (71.40%-90.60%) |  |
| P53wt/P53abn  (n=109, 9.2%) | 20 | 80.90%  (73.26%-88.54%) | 80.90%  (73.26%-88.54%) |  | 15 | 86.90%  (80.24%-93.56%) | 83.90%  (76.26%-91.54%) |  |
| P53abn/P53abn  (n=276, 23.3%) | 91 | 68.50%  (63.01%-73.99%) | 66.10%  (60.22%-71.98%) |  | 70 | 78.40%  (73.5%-83.3%) | 73.10%  (67.61%-78.59%) |  |
| **Abbreviations:** CI, confidence interval; RFS, recurrence-free survival; OS, overall survival; P53wt/P53wt, P53 wild-type in both preoperative biopsy and final pathology; P53abn/P53wt, P53 abnormality in preoperative biopsy while P53 wild-type in final pathology; P53wt/P53abn, P53 wild-type in preoperative biopsy while P53 abnormality in final pathology; P53abn/P53abn, P53 abnormality in both preoperative biopsy and final pathology; a, log-rank test of RFS; b, log-rank test of OS. | | | | | | | | |

**Supplementary table 3**

**AUC of 4-tier P53 expression, classic clinicopathological parameters, and their combination for predicting the recurrence and death of EC**

| **Group** | **AUC (95%CI)** | |
| --- | --- | --- |
|  | **Recurrence** | **Death** |
| 4-tier P53 expression | 0.702  (0.659-0.744) | 0.695  (0.647-0.743) |
| Classic clinicopathological parameters | 0.802  (0.764-0.840) | 0.796  (0.753-0.838) |
| Combination* | 0.856  (0.828-0.885) | 0.838  (0.804-0.871) |
| **Abbreviations:** AUC, area under the curve; Combination*, 4-tier P53 expression+ classic clinicopathological parameters. | | |

**Supplementary table 4**

**Analysis of survival differences between high-risk and non-high-risk groups**

| Group | Number of recurrences  (n=183) | 3-year RFS rate (95%CI) | 5-year RFS rate  (95% CI) | P-value ^a^ | Number of deaths  (n=139) | 3-year OS rate  (95%CI) | 5-year OS rate  (95%CI) | P-value ^b^ |
| --- | --- | --- | --- | --- | --- | --- | --- | --- |
| Non-high-risk group  (n=857, 72.3%) | 45 | 95.10%  (93.53%-96.67%) | 94.40%  (92.83%-95.97%) | <0.001 | 37 | 96.20%  (94.83%-97.57%) | 95.10% (93.53%-96.67%) | <0.001 |
| High-risk group  (n=329, 27.7%) | 138 | 58.80% (53.31%-64.29%) | 54.80%  (48.72%-60.88%) |  | 102 | 72.90%  (68.00%-77.80%) | 64.60% (58.72%-70.48%) |  |
| Abbreviations: CI, confidence interval; RFS, recurrence-free survival; OS, overall survival; a, log-rank test of RFS; b, log-rank test of OS. | | | | | | | | |

**Supplementary table 5**

**Analysis of survival differences of patients receiving different adjuvant therapies in high-risk group.**

| Adjuvant treatment | Number of recurrences | 3-year RFS rate (95%CI) | 5-year RFS rate  (95% CI) | P-value ^a^ | Number of deaths | 3-year OS rate  (95%CI) | 5-year OS rate  (95%CI) | P-value ^b^ |
| --- | --- | --- | --- | --- | --- | --- | --- | --- |
| Follow-up  (N=74) | 39 | 47.30%  (35.54%-59.06%) | 43.70%  (30.96%-56.44%) | 0.038 | 31 | 58.20%  (46.05%-70.35%) | 50.60%  (37.27%-63.93%) | 0.016 |
| Only radiotherapy  (N=103) | 45 | 55.70%  (45.90%-65.50%) | 55.70%  (45.90%-65.50%) |  | 32 | 73.70%  (64.88%-82.52%) | 67.10%  (57.50%-76.70%) |  |
| Only chemotherapy  (N=25) | 12 | 54.90%  (34.91%-74.89%) | 47.00%  (24.85%-69.15%) |  | 10 | 66.00%  (46.60%-85.40%) | 54.50%  (32.94%-76.06%) |  |
| Chemoradiotherapy  (N=127) | 42 | 68.30%  (60.07%-76.53%) | 62.60%  (52.60%-72.60%) |  | 29 | 81.80%  (74.94%-88.66%) | 71.60%  (61.80%-81.40%) |  |
| Abbreviations: CI, confidence interval; RFS, recurrence-free survival; OS, overall survival; a, log-rank test of RFS; b, log-rank test of OS. | | | | | | | | |
